# Distinct Expression Profiles and Different Functions of Odorant Binding Proteins in *Nilaparvata lugens* Stål

**DOI:** 10.1371/journal.pone.0028921

**Published:** 2011-12-09

**Authors:** Peng He, Jin Zhang, Nai-Yong Liu, Ya-Nan Zhang, Ke Yang, Shuang-Lin Dong

**Affiliations:** Education Ministry Key Laboratory of Integrated Management of Crop Diseases and Pests, College of Plant Protection, Nanjing Agricultural University, Nanjing, China; AgroParisTech, France

## Abstract

**Background:**

Odorant binding proteins (OBPs) play important roles in insect olfaction. The brown planthopper (BPH), *Nilaparvata lugens* Stål (Delphacidae, Auchenorrhyncha, Hemiptera) is one of the most important rice pests. Its monophagy (only feeding on rice), wing form (long and short wing) variation, and annual long distance migration (seeking for rice plants of high nutrition) imply that the olfaction would play a central role in BPH behavior. However, the olfaction related proteins have not been characterized in this insect.

**Methodology/Principal Findings:**

Full length cDNA of three OBPs were obtained and distinct expression profiles were revealed regarding to tissue, developmental stage, wing form and gender for the first time for the species. The results provide important clues in functional differentiation of these genes. Binding assays with 41 compounds demonstrated that NlugOBP3 had markedly higher binding ability and wider binding spectrum than the other two OBPs. Terpenes and Ketones displayed higher binding while Alkanes showed no binding to the three OBPs. Focused on *NlugOBP3,* RNA interference experiments showed that *NlugOBP3* not only involved in nymph olfaction on rice seedlings, but also had non-olfactory functions, as it was closely related to nymph survival.

**Conclusions:**

*NlugOBP3* plays important roles in both olfaction and survival of BPH. It may serve as a potential target for developing behavioral disruptant and/or lethal agent in *N. lugens*.

## Introduction

Chemodetection plays a key role in insect behaviors, such as locating food and mates. In insect, most chemosensillums are located on antenna, and others are on mouthparts, wings, legs and ovipositors. The odorant binding proteins (OBPs) in antennae lumen are thought to bind and transport the exogenous odorant to the odorant receptors (ORs) [1], [2], [3], [4], [5], [6], [7] located on dendrite membranes. By interaction between odorant (odorant-OBP complex) and the receptor, cascade reactions are initiated and eventually lead to the transduction of chemical signals to electric signals. After the transduction, the odorant is rapidly inactivated by odorant degrading enzymes (ODEs) to resume the sensitivity of the receptor neurons [8].

OBP is a family of small water soluble proteins consisting of around 120 to 150 amino acids. The six conserved cysteine residues were thought to be the gold standard of an OBP [9]. These six cysteines constitute three disulfide bridges, which together with some amino acids form an odorant binding pocket to bind and protect small hydrophobic ligands [10], [11]. OBP homologues have been identified in numerous species of Orders throughout the Neoptera, which represents more than 98% of all insect species [12], [13], [14]. OBPs are expressed among diverse classes of sensilla, which all have unique odor specificities.

Most OBP binding experiments reported were conducted *in vitro* using recombinant proteins [15], [16], [17], [18]. However, *in vitro* OBP binding with an odor does not necessarily means that the odor is physiologically active because a specific odorant receptor must be present in the same sensillum for the physiological response. In addition, studies in *Drosophila melanogaster* have demonstrated that a muted PBP (the *LUSH^D118A^*) that in configuration closely resembled to the complex of wild PBP (the *LUSH*)/ the sex pheromone (11-*cis* vaccenyl acetate) could activate the receptor [11], suggesting that not just binding but the proper OBP configuration induced by this binding is of primary importance. In addition to the binding assay *in vitro*, an *in vivo* method, the RNA interference (RNAi) was employed to explore the functions of OBPs in insects including *Epiphyas postvittana* [19], *Spodoptera exigua* [20], *Culex quinquefasciatus* and *Anopheles gambiae* [21], [22] and *Drosophila melanogaster* [23]. These studies showed that RNAi is a useful tool in functional studies of genes related to chemosensing.

The brown planthopper (BPH), *Nilaparvata lugens* Stål (Delphacidae, Auchenorrhyncha, Hemiptera), is a notorious rice pest in Asian countries, causing extensive damage by sap-sucking and virus-transmitting. BPH is a monophagous herbivore restricted to cultivated rice and its allied wild rice [24], and therefore a mechanisms to find the rice plants would be crucial in BPH. BPH nymph undergoes 5-6 nymph instars with 3-6 days for each instar, and the adult could live for 15 days or longer [25]. Like many other Delphacidae insects, BPH adults have dual wing form, short wing (brachypterous) and long wing (macropterous). Short wing BPH, unable to fly, occurs when the nature condition (mainly the nutrition quality of rice plants) is suitable for rapid population increase. In contrast, the long wing adults are produced when the population is too high to be sustained by rice plants, and thus inclines to migrate to find rice plants with higher nutritional quality. Additionally, temperature and photoperiodism also affect the wing form. The sensitive stage for wing form change is the 2^nd^-4^th^ instar nymph [26]. BPH conducts annual long distance migrations from tropical areas into subtropical and temperate areas with the help of climate air circulation [27]. The biology of this insect implies a critical role of chemosensory.

Hemiptera, a subgroup of Hemipteroid Assemblage (the sister division to Neoptera Endopterygota) is classified into Sternorrhyncha, Auchenorrhyncha and Heteroptera. So far, research of OBPs in Hemiptera was restricted to few species in Sternorrhyncha such as aphids [28], [29], [30], and in Heteroptera such as *Lygus lineolaris* [31], [32] and *Adelphocoris lineolatus* [18], [33]. For insects in Auchenorrhyncha, OBPs were rarely addressed except for our identification of three putative OBP cDNA fragments in BPH [14] by using ESTs reported by Noda *et al* [34]. In the present study we obtained the full-length cDNA of these OBPs; revealed distinct expression profiles in terms of tissues, development stages, wing forms and genders; obtained ligands binding characteristics; and finally by using RNAi gene silencing combined with behaviour analysis, we demonstrated that *NlugOBP3* plays important roles both in host seeking and in nymph survival in BPH.

## Results

### Full-length cDNAs of *NlugOBPs*

**Figure 1:**
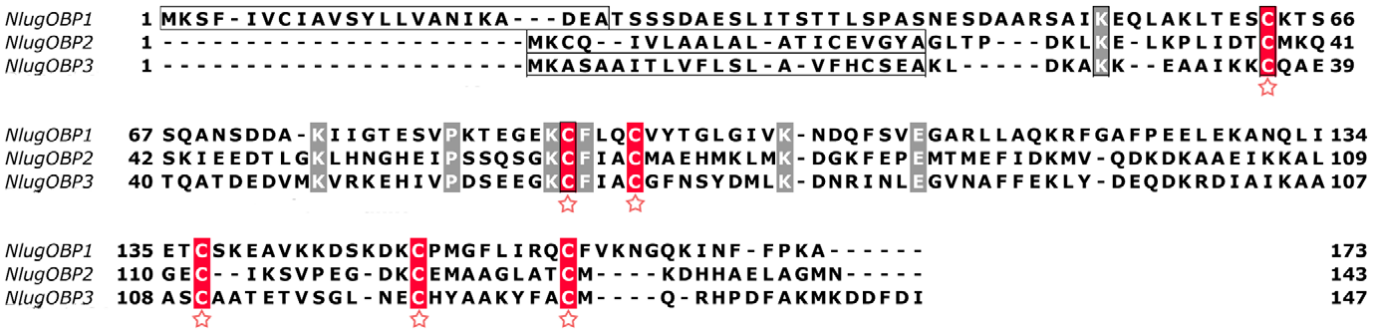
Alignment of amino acid sequences of *NlugOBPs*. Predicted signal peptides were boxed. Conserved cysteines were highlighted in red and marked with ''
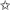
'' below the alignment, and other conserved residues were highlighted in gray.

The cDNA full-lengths of three OBPs were obtained from *N. lugens* by using RACE strategy. These sequences bear all hallmarks of “classic OBP” subfamily, including a signal peptide, a major hydrophobic domain, six conserved Cysteine residues, and considerable identities to reported OBP homologous in amino acid sequence (Table 1 and Fig. 1). *NlugOBP1* had the maximum identity of 46% with an OBP from *Rhodnius prolixus* (CAX63265), whereas the full-length cDNAs of *NlugOBP2* and *NlugOBP3* presented the highest identities with OBP19a (30%) from *D. melanogaster* (NP_728338) and OBP16 (36%) from *Tribolium castaneum* (EFA02853), respectively. However, identities among the three *NlugOBPs* were much lower, only 17% between *NlugOBP1* and *NlugOBP2*, 14% between *NlugOBP1* and *NlugOBP3*, and 24% between *NlugOBP2* and *NlugOBP3*. *NlugOBP1* encoded 173 amino acids (aa), much longer than *NlugOBP2* (143aa) and *NlugOBP3* (147aa).

**Table 1:** Sequence information of three OBP cDNAs cloned from *Nilaparvata lugens.*

Gene name(Gene ID)	ORF(aa)	SP (aa)	3′UTR (bp)	5′UTR (bp)	Blastx result		
					Identity (%)	Species	Protein ID
*NlugOBP1* (FJ215305)	173	23	591	48	46	*R. pro*	CAX63265
*NlugOBP2* (FJ215306)	143	21	593	140	30	*D. mel*	NP_728338
*NlugOBP3* (FJ215307)	147	22	512	68	36	*T. cas*	EFA02853

ORF, open reading frame; SP, signal peptides; UTR, untranslated region; aa, amino acids. *R. pro, Rhodnius prolixus; D. mel, Drosophila melanogaster; T. cas, Tribolium castaneum.*

### Expression profiles of development stage, gender, wing form and tissue

#### Nymphs vs adults

**Figure 2:**
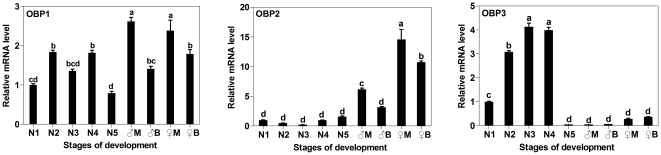
Relative mRNA expression level of *NlugOBPs* in nymphs of different developmental stages, and in adults of different wing forms and genders. The relative expression level was expressed as mean ± SE (N = 3), with the 1^st^ instar nymph as the calibrator. N1-N5, nymph of 1^st^ to 5^th^ instar; ♂M (macropterous) and ♂B (brachypterous), male adults; ♀M and ♀B, female adults.

By qRT-PCR, the expression levels were determined for each OBP with respect to development stages, genders and wing forms (Table 2 and Fig. 2). Generally, *NlugOBP1* in adult showed not much difference in expression level compared with those in nymph. However, *NlugOBP2* expressed significantly higher in adult while in contrast *NlugOBP3* expressed significantly lower than in nymph. *NlugOBP3* expression displayed a sharp decrease in 4^th^ instar compared to 5^th^ instar, therefore, its expression was only partially nymph biased (not including 5^th^ instar).

**Table 2:** Relative expression levels of *NlugOBP* genes in various developmental stages.

	OBP1	OBP2	OBP3
N1	679.34	144.11	840.44
N2	1247.65	72.66	2595.87
N3	922.88	31.71	3468.27
N4	1238.18	142.12	3357.10
N5	533.74	229.13	43.93
♂M	1777.95	890.21	40.53
♂B	959.41	455.40	59.80
♀M	1613.52	2099.75	230.88
♀B	1218.59	1551.02	314.52

N1-N5, nymph of 1^st^ instar to nymph of 5^th^ instar; ♂M, macropterous male; ♂B, brachypterous male; ♀M, macropterous female; ♀B, brachypterous female.

#### Females vs males

**Figure 3:**
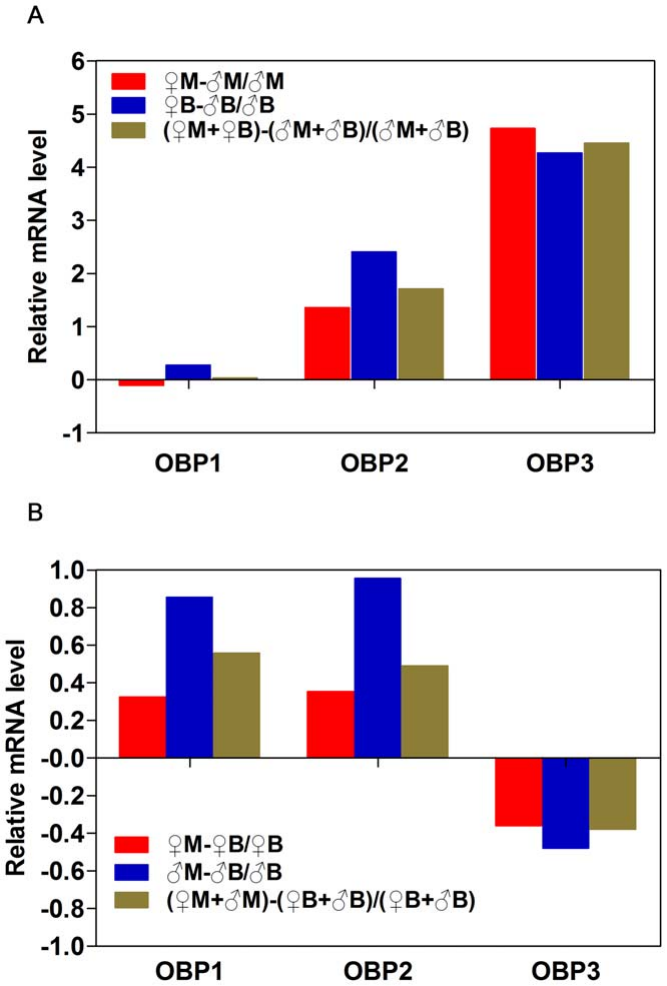
Relative mRNA expression level (♀/♂ or M /B) of OBP genes. (A) For female and male adults, (B) and long and short wing form adults.

Relative expression levels between female and male adults (Fig. 2) were calculated as female / male (IF), and showed in Fig. 3A. *NlugOBP2* and *NlugOBP3* were obviously female biased in both short- and long-wing adults. *NlugOBP1* was expressed in similar levels between females and males.

#### Long wing vs short wing

Similarly, long wing form / short wing form in expression levels were calculated (Fig. 3B). All three *NlugOBPs* showed no great differences between long and short wing adults, with the absolute IF <1.

#### Tissue

**Figure 4:**
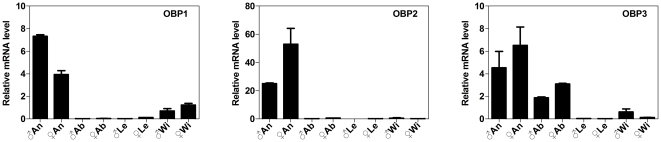
Relative expression levels of *NlugOBPs* in different tissues. The expression levels of *NlugOBPs* were quantified by qRT-PCR. ♂An, male antennae; ♀An, female antennae; ♂Ab, male abdomen; ♀Ab, female abdomen; ♂Le, male legs; ♀Le, female legs; ♂Wi, male wings; ♀Wi, female wings.

*NlugOBP2* expressed high in antenna with little detected in other tissues. *NlugOBP1* expressed high in antennae and also had considerable expression in wings of both sexes. *NlugOBP3* expressed in high levels in antenna and in abdomen. In addition, *NlugOBP1* showed obviously male biased expression, whereas *NlugOBP2* was female biased (Fig. 4).

### *In vitro* expression of OBPs

**Figure 5:**
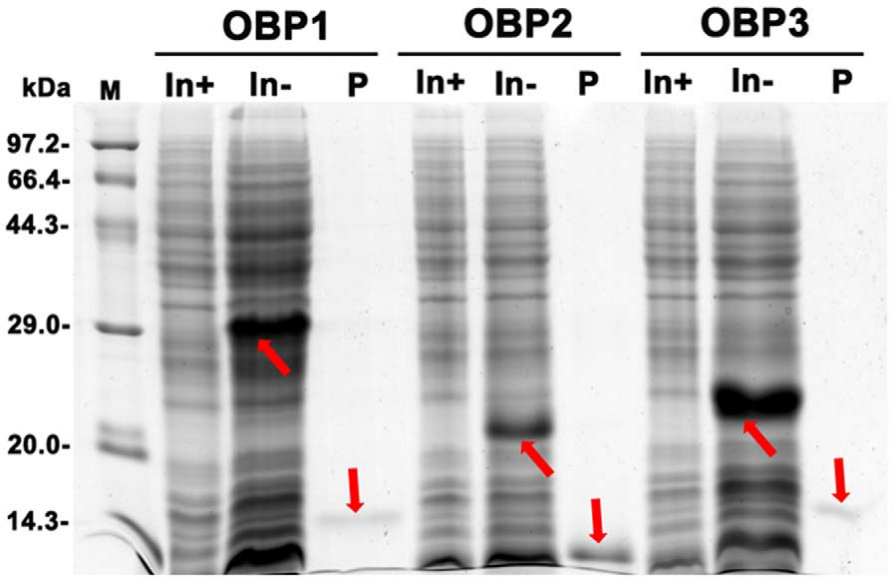
Expression and purification of three NlugOBPs. A SDS-PAGE was used to detect the OBP crude extracts from the bacterial pellets before (In-) and after (In+) induction with IPTG, and purified samples after His-tag cleavage by enterokinase (P). The target bands were marked by arrows.

NlugOBPs were expressed in a bacterial system for ligands binding assays. High yields of recombined proteins (20 mg/L) were achieved. Unlike many other OBPs [15], [16], [30], all three NlugOBPs were not in inclusion bodies but soluble proteins. The purified OBPs were obtained by a His-tag affinity column, and the His-tag was cleavaged by enterokinase (Fig. 5).

### Ligand-binding experiments

**Figure 6:**
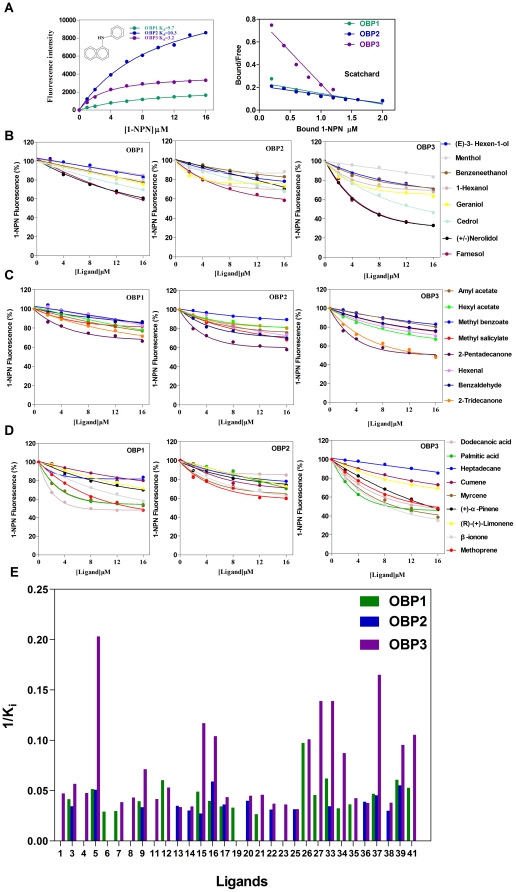
Ligand-binding assay of three NlugOBPs. (A) Binding curves of 1-NPN (Left) and relative Scatchard plot analyses (Right). (B-D) Competitive binding curves of three OBPs with Alcohols (B), Aldehydes, Esters and Benzoates (C), and Carboxylic acids, Alkane and Terpenes (D). (E) Comparison of binding ability (indicated by 1/Ki values) of three OBPs with 31 compounds. Numbers representing ligands were the same as in Table 3 and Table 4.

Fourty-one compounds including 17 rice plant volatiles were selected as ligands to define the binding characteristics of the three NlugOBPs. All of them bound the fluorescent probe (1-NPN) well, with dissociation constants (Kd) of 9.7±0.9, 10.3±1.3 and 3.2±0.3 µM for OBP1, OBP2 and OBP3, respectively (Fig. 6A). Typical displacement curves of 1-NPN by ligands with different function groups were showed in Fig. 6B-D. Based on the displacement curves, IC_50_ and Ki values for all 41 compounds were calculated and listed in Table 3 and Table 4.

**Table 3:** Binding data of recombinant NlugOBPs with different plant volatiles (Alcohols, Aldehydes, Ketones, Esters and Benzoates).

Ligand	Ligand name/	OBP1		OBP2		OBP3	
Number	Structural formula	IC_50_	Ki	IC_50_	Ki	IC_50_	Ki
		(µM)	(µM)	(µM)	(µM)	(µM)	(µM)
	**Alcohols**						
**1**	**1-Hexenol /C_6_H_14_O**	**>50**	**>50**	**>50**	**>50**	**29.7**	**21.2**
**2**	**2-Heptanol/C_7_H_16_O**	**>50**	**>50**	**>50**	**>50**	**>50**	**>50**
**3** *	**(+/-)Nerolidol/C_15_H_26_O**	**28.4**	**24.2**	**33.9**	**29.1**	**24.8**	**17.7**
**4**	**(E)-3-Hexen-1-ol/C_6_H_12_O**	**>50**	**>50**	**>50**	**>50**	**29.6**	**21.1**
**5**	**Farnesol/C_15_H_26_O**	**22.8**	**19.5**	**23.0**	**19.8**	**6.9**	**4.9**
**6**	**Linalool/C_10_H_18_O**	**40.8**	**34.7**	**>50**	**>50**	**>50**	**>50**
**7** *	**2-phenylethanol /C_8_H_10_O**	**39.9**	**34.0**	**>50**	**>50**	**36.6**	**26.1**
**8**	**Geraniol/C_10_H_18_O**	**>50**	**>50**	**>50**	**>50**	**32.6**	**23.3**
**9** *	**Cedrol/C_15_H_26_O**	**30.0**	**25.6**	**35.0**	**30.1**	**19.7**	**14.1**
**10**	**Menthol/C_10_H_20_O**	**>50**	**>50**	**>50**	**>50**	**>50**	**>50**
	**Aldehydes**						
**11** *	**Hexenal/C_6_H_12_O**	**>50**	**>50**	**>50**	**>50**	**33.8**	**24.1**
**12**	**Dodecyl aldehyde/ C_12_H_24_O**	**19.5**	**16.6**	**>50**	**>50**	**26.5**	**18.9**
**13** *	**(E)-2-Hexenal/ C_6_H_10_O**	**>50**	**>50**	**33.5**	**28.8**	**42.0**	**30.0**
**14** *	**Benzaldehyde/ C_7_H_6_O**	**>50**	**>50**	**38.7**	**33.3**	**41.0**	**29.3**
	**Ketones**						
**15** *	**2-Tridecanone/ C_13_H_26_O**	**24.0**	**20.5**	**43.2**	**37.1**	**12.0**	**8.6**
**16** *	**2-Pentadecanone/ C_15_H_30_O**	**29.7**	**25.3**	**19.8**	**17.0**	**13.5**	**9.6**
**17** *	**Acetophenone/ C_8_H_8_O**	**34.3**	**29.3**	**32.3**	**27.8**	**32.4**	**23.1**
	**Esters and Benzoates**						
**18**	**Amyl acetate/ C_7_H_14_O_2_**	**>50**	**>50**	**>50**	**>50**	**>50**	**>50**
**19**	**Isoamyl acetate/ C_7_H_14_O_2_**	**35.7**	**30.5**	**>50**	**>50**	**>50**	**>50**
**20**	**(E)-2-Hexenyl acetate/ C_8_O_2_**	**>50**	**>50**	**29.3**	**25.2**	**31.3**	**22.4**
**21**	**Hexyl acetate/ C_8_H_16_O_2_**	**44.4**	**37.9**	**>50**	**>50**	**30.8**	**22.0**
**22** *	**(Z)-3-Hexenyl acetate/ C_8_O_2_**	**>50**	**>50**	**37.8**	**32.5**	**38.0**	**27.1**
**23** *	**Ethyl benzoate/ C_9_H_10_O_2_**	**>50**	**>50**	**>50**	**>50**	**39.0**	**27.9**
**24**	**Methyl benzoate/ C_8_H_8_O_2_**	**>50**	**>50**	**>50**	**>50**	**>50**	**>50**
**25** *	**Methyl salicylate/ C_8_H_8_O_3_**	**>50**	**>50**	**37.4**	**32.2**	**44.8**	**32.0**

*Marked ligands were identified as rice plant volatile according to literatures; “>50” for the IC_50_ and Ki means that IC_50_ or Ki cannot be accurately calculated with the ligand concentration range tested in the assay.

**Table 4:** Binding data of recombinant NlugOBPs with different plant volatiles (Carboxylic acids, Alkanes, Terpenes and Others).

Ligand	Ligand name	OBP1			OBP2				OBP3			
number	Structural formula	IC_50_	Ki		IC_50_		Ki		IC_50_		Ki	
		(µM)	(µM)		(µM)		(µM)		(µM)		(µM)	
	**Carboxylic acids**											
**26**	**Dodecanoic acid/ C_12_H_24_O_2_**	**12.1**	**10.3**		**>50**		**>50**		**13.9**		**9.9**	
**27** *	**Palmitic acid/ C_16_H_32_O_2_**	**25.9**	**22.1**		**>50**		**>50**		**10.1**		**7.2**	
	**Alkanes**											
**28**	**Eicosane/C_20_H_42_**	**>50**	**>50**		**>50**		**>50**		**>50**		**>50**	
**29**	**Tridecane/C_13_H_28_**	**>50**	**>50**		**>50**		**>50**		**>50**		**>50**	
**30**	**Heptadecane/ C_17_H_36_**	**>50**	**>50**		**>50**		**>50**		**>50**		**>50**	
**31**	**Octadecane/C_18_H_38_**	**>50**	**>50**		**>50**		**>50**		**>50**		**>50**	
**32**	**Tetradecane/ C_14_H_30_**	**>50**	**>50**		**>50**		**>50**		**>50**		**>50**	
	**Terpenes**											
**33** *	**Myrcene/C_10_H_16_**	**19.0**		**16.2**		**33.9**		**29.1**		**10.1**		**7.2**
**34** *	**(+)-α-Pinene/ C_10_H_16_**	**36.4**		**31.1**		**>50**		**>50**		**16.1**		**11.5**
**35** *	**(R)-(+)-Limonene/ C_10_H_16_**	**32.4**		**27.6**		**>50**		**>50**		**33.1**		**23.6**
**36**	**β-Caryophyllene/ C_15_H_24_**	**>50**		**>50**		**30.1**		**25.9**		**37.4**		**26.7**
**37** *	**β- Ionone/C_13_H_20_O**	**25.2**		**21.5**		**25.8**		**22.2**		**8.5**		**6.1**
**38**	**Cumene /C_9_H_12_**	**>50**		**>50**		**39.2**		**33.7**		**37.2**		**26.6**
**39**	**Methoprene/C_19_H_34_O_3_**	**19.4**		**16.6**		**21.1**		**18.1**		**14.7**		**10.5**
	**Others**											
**40**	**Cineole/C_10_H_18_O**	**>50**		**>50**		**>50**		**>50**		**>50**		**>50**
**41**	**2,6-Di-tert-butylphenol/ C_14_H_22_O**	**22.3**		**19.0**		**>50**		**>50**		**13.3**		**9.5**

*Marked ligands were identified as rice plant volatile according to literatures; “>50” for the IC_50_ and Ki means that IC_50_ or Ki cannot be accurately calculated with the ligand concentration range tested in the assay.

In general, Ketone and Terpene presented high binding affinities to the OBPs. For example, 2-Tridecanone, 2-Pentadecanone, Myrcene, (+)-α-Pinene, β-Ionone and Methoprene showed very high affinities with NlugOBP3 (Ki <12). In contrast, Alkanes showed no binding ability with OBPs, with incalculable Ki. Differences among the three OBPs were also found. Firstly, OBP1 and OBP2 could bind only 19 and 15 ligands, respectively, while OBP3 could bind 28 ligands indicating a broader binding spectrum of OBP3. Secondly, OBP3 showed higher binding abilities than OBP1 and OBP2, based on IC_50_ and Ki values. The averaged IC_50_ (25.3) and Ki (18.7) of OBP3 were obviously lower than those of OBP1 (IC_50_, 28.2; Ki, 24.0) and OBP2 (IC_50_, 27.1; Ki, 24.6). However, Linalool and Isoamyl acetate were two exceptions, exhibited some affinities with NlugOBP1, but none with OBP3 (Fig. 6, Table 3 and Table 4).

### Functional investigation of *NlugOBP3*

**Figure 7:**
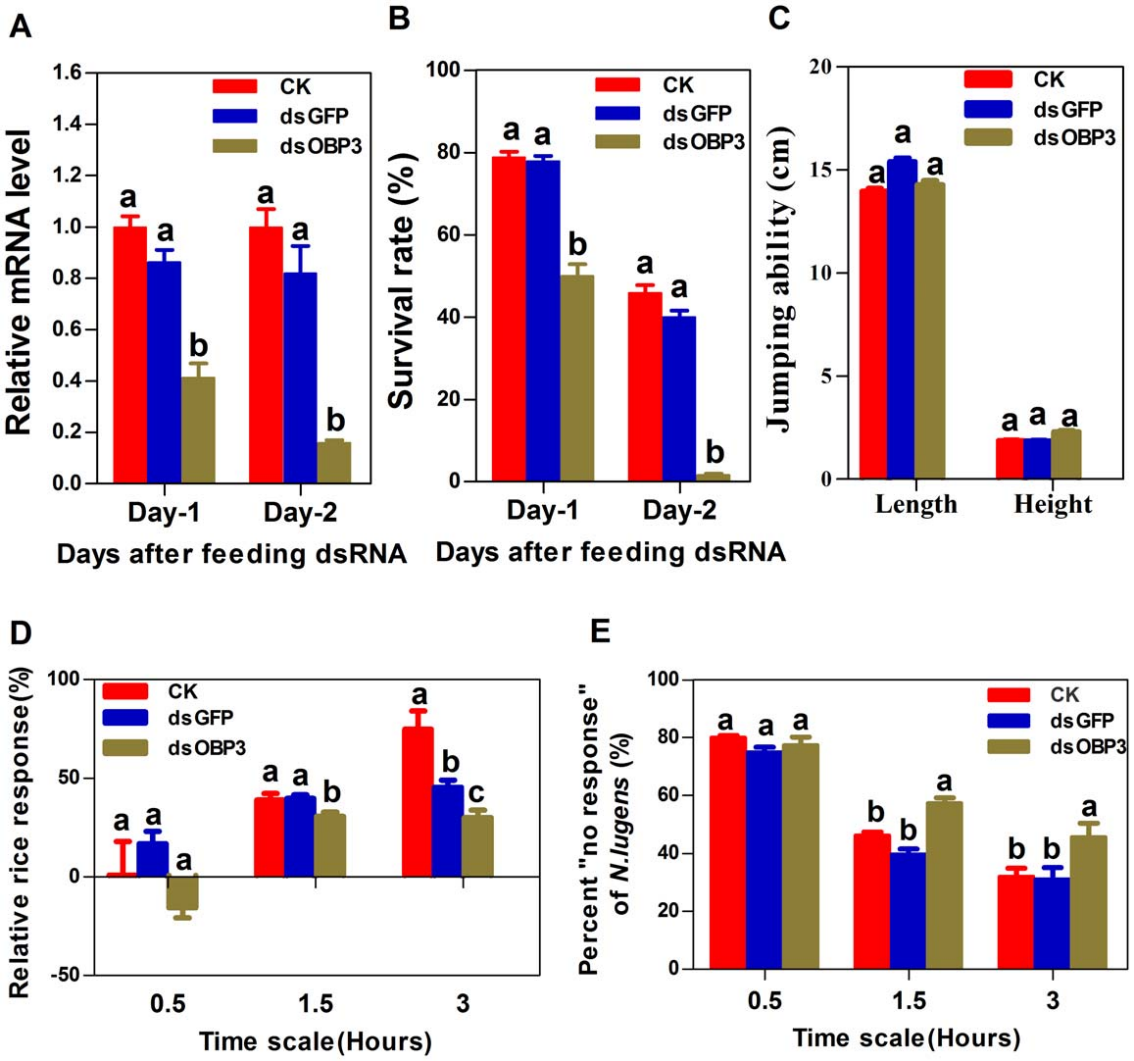
Effects of feeding *NlugOBP3* dsRNA on NlugOBP3 mRNA level (A), survival rate (B), jumping ability (C), and response to rice seedlings (D-E) of BPH nymphs. CK, nymph fed with normal diet; dsGFP, fed with diet mixed with dsRNA of green fluorescent protein (0.5 mg/ml); dsOBP3, fed with dsRNA of *NlugOBP3* (0.5 mg/ml). Data topped with different letters are significant different as determined using a one-way ANOVA (Duncan’s multiple range test, P<0.05). All error bars represent the SE of the mean obtained from at least three independent replicates.

As indicated by above experiments, *NlugOBP3* was the most expressed one in the nymph stage among the three OBPs, and showed a wider binding spectrum and higher binding affinities compared to the other two. Furthermore, *NlugOBP3* expression was significantly nymph biased, but with a sharp decrease in 5^th^ instar nymph, implying a possible role in BPH metamorphosis as well as in olfaction. *NlugOBP3* was therefore selected for the *in vivo* function study by RNAi. Comparing to the insects feeding on a normal artificial diet (CK) and an exogenous dsRNA mixed diet (dsGFP), the nymph on OBP3-dsRNA diet (dsOBP3) showed a significantly reduced mRNA expression, with about 60% and 80% reduction at 1 day and 2 days after the treatment, respectively (Fig. 7A).

Very surprisingly, the survival rate of dsOBP3 treated nymphs was significantly decreased compared to the CK and dsGFP groups. Only 50% survival rate was observed in dsOBP3 group, while about 80% survival rates were showed in dsGFP and CK groups at 1 day after the treatment. At 2 days after the treatment, <5% survival rate was observed in dsOBP3 group (Fig. 7B). To exclude the possibility that the high mortality in dsOBP3 group was resulted from the reduced nymph vitality, a jumping assay was conducted. The results showed no difference both in jumping distance and jumping height between the dsOBP3 group and the two control groups (Fig. 7C).

Two-choice bioassays revealed that the seeking behavior of second instar nymphs for rice seedlings was significantly inhibited by OBP3 dsRNA diet (Fig. 7D). At 1.5 h and 3.0 h after placed into the olfactometer, the relative response (%) was significantly lower in dsOBP3 group than that in CK and dsGFP groups. Meanwhile, the non-response rate of dsOBP3 group was significantly higher than that of CK and dsGFP groups at 1.5 and 3.0 h.

## Discussion

### The amino acids sequences of OBPs in BPH

Based on the fragments obtained in our previous work [14], full-length cDNAs of the three OBPs of BPH were cloned in the present study. This is the first report of this kind, providing the basis for elucidation of mechanisms underlying chemosensing in BPH. The OBPs in BPH have conserved features of typical OBPs such as the six cyseines and some amino acid residues. The low similarity (<25%) among the three *NlugOBPs* also was consistent with that in other insects [30], [33], [35], which suggests that these OBPs diverged at early time or/and are still evolving.

### OBP expression patterns and functional implications

Expression patterns of tissues, developmental stages, sexes and other specific phenotypes can provide functional clues of genes. All three *NlugOBPs* were expressed more in antennae than in other tissues (Fig. 4), suggesting an olfactory role of these OBPs. In addition, *NlugOBP1* also expressed a considerable amount in wings (Fig. 4A, B), therefore it may also involve in gustatory function, as wing plays somewhat gustory role in insects.

*NlugOBP3* may have functions besides olfaction as it expressed highly in the non-olfactory abdomen (Table 2 and Fig. 4). Further investigation on the temporal expression showed that OBP3 displays a nymph (before 5^th^ instar) biased expression with a 4-fold reduction from 4^th^ instar to 5^th^ instar (Table 2 and Fig. 2). Since 5^th^ instar is the final nymph stage before molting to adults, this temporal expression pattern may suggest its involvement in metamorphosis in addition to olfactory function, which is confirmed by the RNAi experiment and subsequent bioassay (see detailed discussion below).

### Binding ability of NlugOBPs with odors of different structures

Locating host plant is essential for a phytophagous insect [36]. An efficient mechanism of host detection and recognition is particularly important for BPH as it is a monophagous and a long distance migrating insect. The competitive binding experiments showed that the three NlugOBPs displayed some common binding features and also some differences (Table 3, Table 4 and Fig. 6).

Four volatiles of rice plants (2-Tridecanone, 2-Pentadecanone and β-Ionone, and Acetophenone) are present in a volatile mixture that is attractive to BPH [37], [38]. In our experiments, these four volatiles also showed relatively high binding abilities to all three *NlugOBPs*, especially to *NlugOBP3* (Ki<12, except for Acetophenone) (Fig. 6C, D, Table 3 and Table 4). Two green leaf volatiles (6-carbon compounds) Hexanal and (E)-2-Hexenal were among the most abundant volatiles of rice [39], and generate high Electroantennogram (EAG) response in BPH and some other Hemiptera insects [40], [41], [42]. As expected, NlugOBP3 could bind these two volatiles, although the Ki were not very high. However, NlugOBP2 can bind only with (E)-2-Hexenal; and NlugOBP1 binds none of the two volatiles (Fig. 6E and Table 3).

Another group of volatiles showing high affinity with the three OBPs is Terpenes. These volatiles (e.g. α-Pinene, β-Pinene, Myrcene, Limonene, Camphene and Carene) attract a number of beetle species [43]. α-Pinene and Limonene are components of rice plant volatiles [39], [44]. In our study, Myrcene and α-Pinene showed extremely high binding abilities with NlugOBP3 (Ki  = 7.2 and 11.5 µmol/L, respectively) (Table 4). Limonene and β-Caryophyllene also showed considerable affinities with NlugOBP3 (Fig. 6D, E). These volatiles could be a valuable resource to identify attractants or deterrents for the control of BPH as well as other insect pests.

A very interesting finding in our binding assay is that Farnesol and Methoprene, two juvenile hormone analogues of insects [45], [46], have high binding ability with all three OBPs, especially with NlugOBP3 (Fig. 6B, D and Table 4). Combining with the expression profile (high abdomen expression, and 1^st^-4^th^ instar nymph and female biased expression), we could hypothesize that OBP3 is involved in transporting juvenile hormone in BPH. The later RNAi experiments showing high mortality of OBP3 deficit nymphs partially supported this hypothesis. The possible role of OBP in juvenile hormone transport deserves further investigations.

Additionally, NlugOBP3 has obviously broader binding spectrum and higher binding ability with tested volatiles compared to NlugOBP1 and NlugOBP2. Taken together with its distinct expression profile and higher expression level, OBP3 probably is more important than the other two OBPs in odor perception and/or other functions.

### Effects of RNAi of *NlugOBP3*

RNAi is widely used to investigate gene functions in insects as well as in other organisms, and the technology has been successfully used in BPH gene silencing using dsRNA diet or injection [47], [48]. As discussed earlier, *NlugOBP3* possibly has functions in addition to olfaction. To confirm this, *NlugOBP3* RNAi diet experiments were conducted. The results showed that target gene silencing rate is very high, up to about 60% at one day and about 80% at two days after treatment (Fig. 7A), providing an *in vivo* methodology for OBP gene silencing in BPH.

The RNAi experiments and two-choice olfactometer bioassays together revealed that *NlugOBP3* plays a role in perception of rice plant volatiles, as the host-seeking behavior of nymphs was significantly inhibited after silencing *NlugOBP3* expression (Fig. 7D). However, the “no response” nymphs in dsOBP3 group were also significantly increased compared to the non-dsRNA control groups. The jumping assay excluded the possibility that the reduced response rate of dsOBP3 treated nymphs is due to low viability (Fig. 7C), thus confirmed that the lower response rate is due to the reduced olfaction of the seedling volatiles.

More importantly, our RNAi experiment revealed the non-olfactory function of NlugOBP3, as silencing the gene resulted in strikingly high nymph mortality (Fig. 7B). The possibility that this mortality was induced by olfaction suppression can be excluded because the artificial liquid diets used in the RNAi experiment contain no plant volatiles. As speculated earlier, the high mortality may due to the disruption of JH binding and transporting possibly carried out by *NlugOBP3.*

To summarize, we obtained the full-length cDNAs coding for three OBPs in BPH for the first time and conducted experiments on expression patterns, ligand binding properties and phenotype changes after gene silencing (*NlugOBP3* only). The results suggest that *NlugOBP3*, compared to *NlugOBP1* and *NlugOBP2*, plays more important roles in olfaction of plant volatiles, and may possess non-olfactory functions associated with BPH survival. *NlugOBP3* could be a potential target for behavioral disruptant and lethal agent for BPH.

## Materials and Methods

### Insect rearing and collection

BPH were collected from a rice field at Jiangsu Academy of Agricultural Science, China, and reared with rice seedlings in plastic box (40×30×40 cm) in the laboratory under 26±1°C, 16 h light : 8 h dark cycle and 70–90% relative humidity. Nymphs of different instars and virgin long and short wing adults of both sexes were collected in three replications (20 individuals per replication). Various tissues from virgin short wing adults were dissected under microscope and collected in three replications for each tissue type. Each replication contained 100 antenna or wings, or 40 legs, or 20 abdomen. Whole body and tissue samples were all frozen in liquid nitrogen and stored at -70°C until use.

### RNA isolation and cDNA synthesis

Total RNA was extracted by SV 96 Total RNA Isolation System (Promega, Madison, WI, USA) following the manufacturer's instructions. RNA quality was checked with a spectrophotometer (NanoDrop™ 1000, Thermo Fisher Scientific, USA). The single-stranded cDNA templates were synthesized using 1 µg total RNAs from various samples with oligo (dT) 18 primer as the anchor primers. The M-MLV Reverse Transcriptase (M-MLV) (TaKaRa, Dalian, Liaoning, China) was used for the cDNA synthesis, with reaction conducted at 42°C for 1 h, and then stopped by heating at 70°C for 15 min.

### RACE amplification and sequence alignment

To get the full-length sequence of OBPs, Rapid Amplification of cDNA End (RACE) PCR was performed using GeneRacer™ kit (Invitrogen Carlsbad, CA, USA) for 3′end and 5′ends amplification. The full-length sequences were assembled with RACE results and then confirmed by end-to-end PCR using specific primers designed at both ends. Three positive clones of each gene were sequenced to detect possible PCR mistakes. The primer sequences designed by Primer Premier 5.0 (PREMIER Biosoft International, CA, USA) were listed in Table S1. Multiple alignments of OBPs protein sequences were performed using CLUSTALX 2.0 [49] and arranged by Jalview 2.4.0 b2 [50]. The signal peptides were predicted by SignalP 3.0 http://www.cbs.dtu.dk/services/SignalP/) [51].

### Quantitative RT-PCR

Before quantitative RT-PCR (qRT-PCR), normal RT-PCR were done with each primer pair to ensure that the correct products were being amplified and no primer dimer was present using rTaq DNA polymerase (TaKaRa, Dalian, Liaoning, China) and 1.5% (w/v) electrophoresis agarose gel. The qRT-PCR reactions for each sample were performed on an ABI 7300 (Applied Biosystems, Foster City, CA, USA) using 20 ng of cDNA template and 10 µM gene-specific primers designed by Beacon Designer 7.6 (PREMIER Biosoft International, CA, USA), which were listed in Table S1. The reactions were 10 s at 95°C, followed by 40 cycles of 95°C for 5 s and 60°C for 31 s. The mRNA levels were measured by qRT-PCR using the SYBR *Premix Ex Taq*™ (TaKaRa, Dalian, Liaoning, China). This was followed by the measurement of fluorescence during a 55 to 95°C melting curve in order to detect a single gene-specific peak and to check the absence of primer dimer peaks. Under these conditions, a single and discrete peak was detected for all primers tested. Negative controls were non-template reactions (replacing cDNA with H_2_O). Ten-fold dilution series were used to construct a relative standard curve to determine the PCR efficiencies and for further quantification analysis. In all experiments, all primers gave amplification efficiencies of 90-100%. Each reaction was run in triplicate (technical replicate). mRNA level was quantified in relation to the expression of β-actin (EU179846 [52] ) that was used as the control gene. The primer pair for each gene was designed to amplify a 70-200 bp product, which was verified by nucleotide sequencing. Means and standard errors were obtained from the average of three independent biological replicates. The relative copy numbers of OBP genes were calculated according to the 2^−ΔΔCt^ method [53]. The mRNA level in different samples was analyzed using the ABI 7300 analysis software SDS 1.4.

### *E. coli* expression and purification of the recombinant protein

The three *NlugOBP* sequences encoding mature proteins were amplified by primers including BamH I and Xho I restriction enzyme sites (Table S1). The purified PCR products were ligated into pEASY T3 cloning vector (TransGen, Beijing, China) for 15 min, and then the products were transformed to *E.coli* Trans1-T1 competent cells. Three positive clones were sequenced by Genscript Biology Company (Nanjing, Jiangsu, China). The plasmids contained appropriate OBP sequences and the empty pET30a plasmid were digested with BamH I and Xho I FastDigest® restriction enzymes (Fermentas, Thermo Fisher Scientific, USA) for 10 min at 37°C. The purified double-enzymes digested products of OBPs were ligated into digested empty pET30a with T4 ligase (Fermentas, Thermo Fisher Scientific, USA) for 1 h at 22°C. After ligation, the products were transformed into BL-21 DE3 pLys *E. coli* cells. The positive clones were validated by PCR and sequencing. The expression of recombinant proteins were induced by addition of IPTG to a final concentration of 2 mM when the LB medium culture had reached a value of OD600 = 0.5. Cells were grown overnight with 200 rpm and 37°C, harvested by centrifugation (8000 g×20 min 4°C) and sonicated. All the protein was dissoluble. The proteins were purified by XK-16 Column with Ni Sepharose High performance (GE Healthcare Life Sciences). The His-tag was cleavaged by enterokinase (Genscript Biology Company, Nanjing, China). The cleavaged proteins were purified again by column mentioned above with naturing agent. The purified proteins without His-tag were desalinated by dialysis against NaCl. The resulted proteins were kept at -70°C after freeze-dry.

### Competitive fluorescence binding assay

To measure the affinity of OBPs to the fluorescent probe N-phenyl-1-naphthyl-amine (1-NPN), a 2 M solution of the protein in 50 mM Tris–HCl, pH 7.4, was titrated with aliquots of 1 mM ligand in methanol to achieve various concentrations. The affinity to other ligands was measured in competitive binding assays, using both the protein and the fluorescent reporter 1-NPN at 2 µM concentration. The excitation wavelength was 337 nm and the emission spectrum was recorded between 370 and 460 nm by a fluorescence spectrometer (Hitachi F-7000 Fluorescence Spectrophotometer, Japan) with a 1 cm light path quartz cuvette and 10 nm slits for both excitation and emission. Dissociation constants for 1-NPN were calculated from Scatchard plots of the binding data, and the relative to other ligands were calculated from the corresponding IC_50_ values, using the equation: Ki  =  [IC_50_]/1+ [1-NPN]/K_1-NPN_, where [1-NPN] is being the free concentration of 1-NPN and K_1-NPN_ being the dissociation constant of the complex protein/1-NPN.

### Double strand RNA synthesis

The full cDNA sequence *NlugOBP3* (FJ215307) and partial sequence of a green fluorescent protein (GFP ACY56286) were sub-cloned into pEASY-T3 vector, and the dilution plasmid was used as template for amplification of the target sequences. The sequence of *NlugOBP3* was amplified by RT-PCR using specific primers conjugated with 23 bases of the T7 RNA polymerase promoter (Table S1).The PCR products of 487 bp for *NlugOBP3* and 460 bp for GFP were purified using Wizard® SV Gel and PCR Clean-Up System (Promega, Madison, WI, USA) and used as templates for dsRNA synthesis using the T7 Ribomax^TM^ Express RNAi System (Promega, Madison, WI, USA). The synthesized dsRNA was isopropanol precipitated, resuspended in Nuclease-free water, and quantified by a spectrophotometer (NanoDrop™ 1000, Thermo Fisher Scientific, USA) at 260 nm. The purity and integrity were determined by agarose gel electrophoresis. It was kept at -70°C until use.

### Double strand RNA diet and gene expression analysis

dsRNA rearing procedure reported by Fu *et al* [25] and Chen *et al* [48] was used with small modifications. Briefly, nymphs of second instar that were pre-reared on artificial diets for one day were used. Glass cylinders, 12 cm in length and 2.8 cm in internal diameter, were used as feeding chambers. Twenty nymphs were carefully transferred into each chamber. The dsRNA was added to the artificial diet at 0.5 µg/µl [48]. This mixed diet (40 µl) was held between two layers of stretched Parafilm M (Pechiney Plastic Packaging Company, Chicago, IL, USA) that was placed at both ends of the chamber. One more piece of Parafilm M was covered to avoid entering of hydrosphere which may stick the nymphs to the cylinder wall. The chamber bodies were covered with black and wet cotton cloth exposing only the two ends (the diet) to the light. Fresh diet was provided daily and dead nymphs were cleaned.

Three treatments including non-dsRNA diet (CK), GFP dsRNA diet (dsGFP) and OBP3 dsRNA diet (dsOBP3) were set up and replicated six times (6 chambers). The experiment last for two days. On each day, one nymph from each chamber (totally 6 nymphs) was collected. Two nymphs were designed as one replicate (3 replicates for each treatment at each sampling time) for RNA isolation. The qRT-PCR method and reaction conditions were same as described above. Primers used in qRT-PCR were listed in Table S1.

The mortality effect of dsRNA diet on nymphs was evaluated in a separate experiment, where the method was the same as the dsRNA feeding experiment, but replicated 8 times (8 chambers). Mortality was recorded on both days.

### Nymph vitality bioassay

To test the possible influence of dsRNA feeding on BPH vitality, a jumping ability assay was conducted at one day after the feeding. One BPH nymph (2^nd^ instar) was placed in a glass cylinder (same as above) with both ends sealed by Parafilm. Holding vertically with one hand, the bottom end was gently knocked by a finger to induce active jumping. The heights of the jumps were recorded. The jumping distance (length) was measured by stimulating a nymph in an open dish with a gently touch at the abdomen using an insect pin. Each nymph was stimulated 2-4 times in jumping height or length measurement and the averages were used. Thirty nymphs were tested for each treatment group.

### Two-choice behavioral bioassay

The behavioral responses of BPH to rice volatiles was tested by a two-choice bioassay using an H-shaped olfactometer similar to that used by Khan and Saxena [54] and Lou and Chen [55] (Fig. S1). The olfactometer mainly comprises of two glass tubes (arms) (10 cm diameter×30 cm long) with gauze at its top end connected at their center point by another smaller glass tube (8 cm diameter×20 cm long) with nylon mesh at two ends and a small hole (1 cm diameter) at its half way point for releasing BPH.

About 20 g fresh rice seedlings with no insect damage were caged in one arm of the olfactometer. Twenty nymphs (2^nd^ instars) were introduced, and then the hole was sealed with absorbent cotton. BPH in “B” and “C” area were counted at 0.5, 1.5 and 3.0 h.

Three treatments of BPH nymphs (one day after dsOBP3 feeding, one day after dsGFP feeding, and non-dsRNA feeding CK) were tested in four replications. After 2 tests (replications), the olfactometers were washed with 75% alcohol and the rice seedlings were placed in another arm to complete the other two replicates. At any given time, nymphs in two treatments were tested using two olfactometers. Totally 12 bioassays were performed in two days in a darkroom at (26±1)° and 70–90% relative humidity.

The relative response percentages (RRP) were calculated as[(number of nymph responded to rice seedlings (NRC) – numbers of nymph responded to control air (NRA)/(NRC+NRA)]×100%.

### Data analysis

All data (mean ± SE) were compared with one-way nested analysis of variance (ANOVA Duncan's multiple range test) using SPSS Statistics 18.0 (SPSS Inc., Chicago, IL, USA).

## Supporting Information

Figure S1 Schematic diagram of H-shaped olfactometer used for behavioral assay. A, Release hole; B, The area defined as response to rice seedlings; C, The area defined as response to air; D, Pot with rice plants; E, Pot with nothing.
(TIF)

Table S1 Primers used in RACE, qRT-PCR, Vector construction and dsRNA synthesis.
(DOC)
