# Supplementary figures and images for: Distinct Expression Profiles and Different Functions of Odorant Binding Proteins in Nilaparvata lugens Stål
Source: PLoS One. 2011 Dec 9;6(12):e28921. doi: 10.1371/journal.pone.0028921 (PMC3235172; doi:10.1371/journal.pone.0028921)

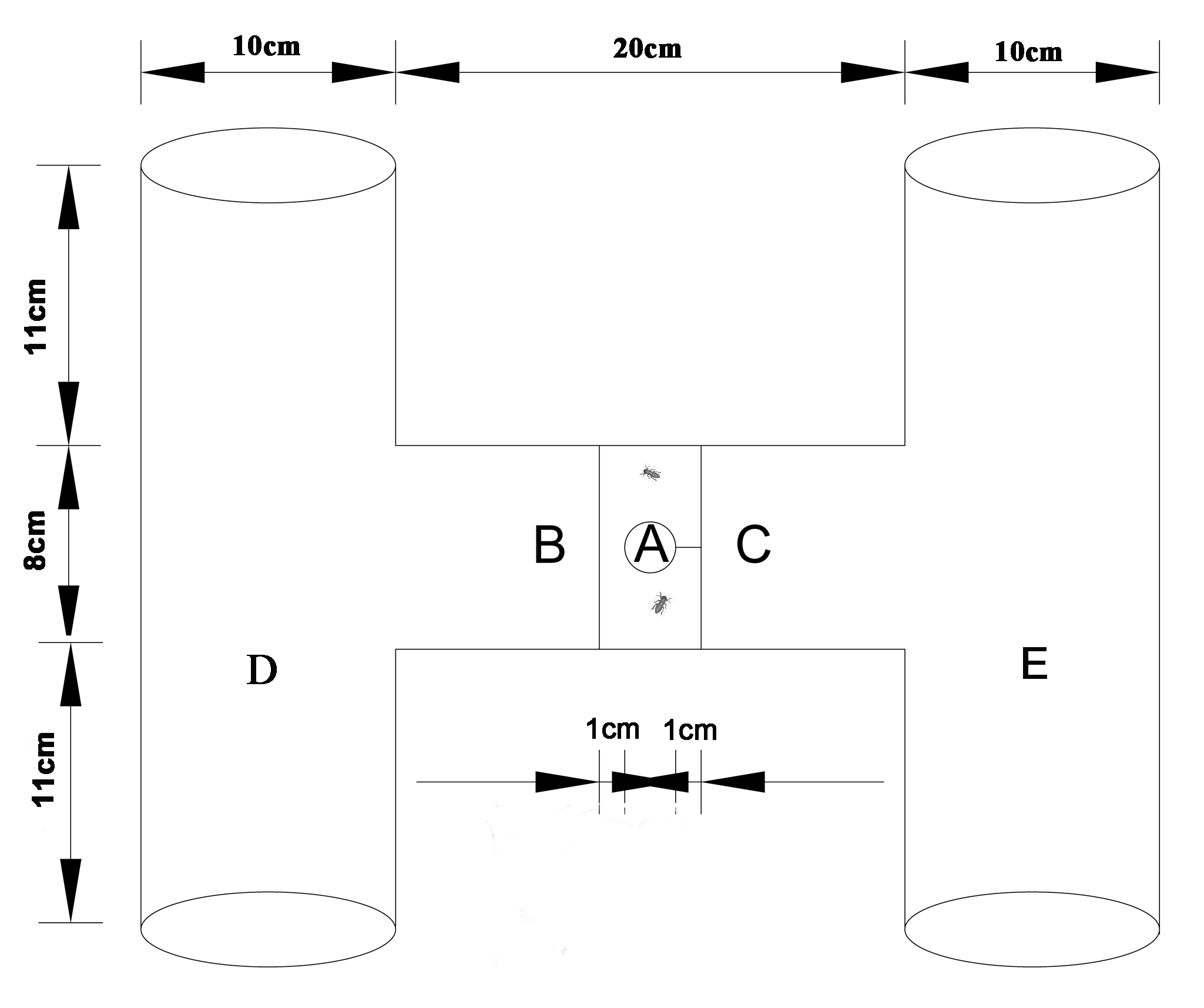

Supplement: Figure S1 — Schematic diagram of H-shaped olfactometer used for behavioral assay. A, Release hole; B, The area defined as response to rice seedlings; C, The area defined as response to air; D, Pot with rice plants; E, Pot with nothing. (TIF) [file pone.0028921.s001.tif]
