# Supplementary material for: Distinct Expression Profiles and Different Functions of Odorant Binding Proteins in Nilaparvata lugens Stål
Source: PLoS One. 2011 Dec 9;6(12):e28921. doi: 10.1371/journal.pone.0028921 (PMC3235172; doi:10.1371/journal.pone.0028921)
Supplement: Table S1 — Primers used in RACE, qRT-PCR, Vector construction and dsRNA synthesis. (DOC) [file pone.0028921.s002.doc]

**Table S1 Primers used in RACE,** **qRT-PCR**, **Vector construction and dsRNA synthesis**

| Purpose | Primer name | Sequence (5’→3’) |
| --- | --- | --- |
| RACE PCR | NlugOBP1-5’ | gaattggcttggctggaggttttgca |
|  | NlugOBP1-3’ | cgcggccaggtctgccatcaa |
|  | NlugOBP2-5’ | catatgttcagccatgcaggcaataaagca |
|  | NlugOBP2-3’ | atggccacgagattccaagctcacaatc |
|  | NlugOBP3-5’ | caggatgcctctgcatacaggcgaagta |
|  | NlugOBP3-3’ | cccaagccactgacgaggatgtaatgaa |
| qRT-PCR | NlugOBP1-qF | tttggcacagaaacgatttggag |
|  | NlugOBP1-qR | cattgggcacttgtctttggag |
|  | NlugOBP2-qF | catcaagagtgtaccagaaggagac |
|  | NlugOBP2-qR | aatcatcagttcataccagcaagc |
|  | NlugOBP3-qF | aagccactgacgaggatgtaatg |
|  | NlugOBP3-qR | ttcacaccttccaagttgattctg |
| Vector construction | NlugOBP1-vF | atggatccgacgaggcaacatcttcatcag |
|  | NlugOBP1-vR | gatctcgagttaggctttaggaaagaagtttatcttc |
|  | NlugOBP2-vF | atggatccggactcacaccagacaaattga |
|  | NlugOBP2-vR | gatctcgagtcagttcataccagcaagctca |
|  | NlugOBP3-vF | atggatccaagcttgacaaagccaagaagg |
|  | NlugOBP3-vR | gatctcgagctagatatcgaagtcgtccttcatct |
| dsRNA synthesis | NlugOBP3-dsF | taatacgactcactatagggatgaaggcttcagctgctat |
|  | NlugOBP3-dsR | taatacgactcactataggggatatcgaagtcgtccttca |
|  | GFP-dsF | taatacgactcactatagggaagttcagcgtgtccg |
|  | GFP-dsR | taatacgactcactatagggcaccttgatgccgttc |
